# Pharmacology of Kappa Opioid Receptors: Novel Assays and Ligands

**DOI:** 10.3389/fphar.2022.873082

**Published:** 2022-04-21

**Authors:** Chiara Sturaro, Davide Malfacini, Michela Argentieri, Francine M. Djeujo, Erika Marzola, Valentina Albanese, Chiara Ruzza, Remo Guerrini, Girolamo Calo’, Paola Molinari

**Affiliations:** ^1^ Department of Neuroscience and Rehabilitation, Section of Pharmacology, University of Ferrara, Ferrara, Italy; ^2^ Department of Pharmaceutical and Pharmacological Sciences, University of Padova, Padova, Italy; ^3^ Department of Chemical, Pharmaceutical and Agricultural Sciences, University of Ferrara, Ferrara, Italy; ^4^ Technopole of Ferrara, LTTA Laboratory for Advanced Therapies, Ferrara, Italy; ^5^ National Center for Drug Research and Evaluation, National Institute of Health, Rome, Italy

**Keywords:** kappa opioid receptor, G protein-coupled receptor, label-free, BRET, calcium mobilization, biased agonism, PWT2-dyn A, dyn A-palmitic

## Abstract

The present study investigated the *in vitro* pharmacology of the human kappa opioid receptor using multiple assays, including calcium mobilization in cells expressing chimeric G proteins, the dynamic mass redistribution (DMR) label-free assay, and a bioluminescence resonance energy transfer (BRET) assay that allows measurement of receptor interaction with G protein and β-arrestin 2. In all assays, dynorphin A, U-69,593, and [D-Pro^10^]dyn(1-11)-NH_2_ behaved as full agonists with the following rank order of potency [D-Pro^10^]dyn(1-11)-NH_2_ > dynorphin A ≥ U-69,593. [Dmt^1^,Tic^2^]dyn(1-11)-NH_2_ behaved as a moderate potency pure antagonist in the kappa-β-arrestin 2 interaction assay and as low efficacy partial agonist in the other assays. Norbinaltorphimine acted as a highly potent and pure antagonist in all assays except kappa-G protein interaction, where it displayed efficacy as an inverse agonist. The pharmacological actions of novel kappa ligands, namely the dynorphin A tetrameric derivative PWT2-Dyn A and the palmitoylated derivative Dyn A-palmitic, were also investigated. PWT2-Dyn A and Dyn A-palmitic mimicked dynorphin A effects in all assays showing similar maximal effects but 3–10 fold lower potency. In conclusion, in the present study, multiple *in vitro* assays for the kappa receptor have been set up and pharmacologically validated. In addition, PWT2-Dyn A and Dyn A-palmitic were characterized as potent full agonists; these compounds are worthy of further investigation *in vivo* for those conditions in which the activation of the kappa opioid receptor elicits beneficial effects e.g. pain and pruritus.

## Introduction

The kappa opioid receptor was cloned in 1993 (Meng et al., 1993), while its pharmacological identification spans more than two-decades before. Portoghese and others proposed more than a single opioid receptor type existed (Portoghese, 1965). The earliest direct demonstrations of multiple opioid receptor binding sites were obtained with radiolabelled naloxone and etorphine molecules (Pert and Snyder, 1973; Simon et al., 1973; Terenius, 1973). The first definitive evidence that these receptors did not form a homogeneous population was presented in 1976 (Martin et al., 1976), and kappa opioid receptors as specific subtype confirmed with structure-activity relationship studies on the dynorphin(1-13) scaffold (Chavkin and Goldstein, 1981). Subsequent medicinal chemistry and molecular biology studies identified major ligand binding and receptor domains (Hjorth et al., 1995; Hjorth et al., 1996; Thirstrup et al., 1996; Cheng et al., 1998; Coward et al., 1998; Jones et al., 1998; Bonner et al., 2000; Celver et al., 2002; Owens and Akil, 2002; Zhang et al., 2002; Pascal and Milligan, 2005; Yan et al., 2005): discoveries recently confirmed via X-ray crystallography (Wu et al., 2012; Che et al., 2020).

Kappa opioid receptor is a G protein-coupled receptor (GPCR), whose activation mediates guanosine diphosphate (GDP) to guanosine triphosphate (GTP) exchange in the alpha subunit of pertussis toxin-sensitive heterotrimeric G proteins. These G proteins dissociate and activate downstream cascades, including depression of cyclic adenosine monophosphate (cAMP) formation, opening of potassium and closing of voltage-gated calcium channels. Activated kappa opioid receptors interact with G protein-coupled receptor kinases (GRK: GRK2,3,5,6, (Chen et al., 2016; Chiu et al., 2017)) leading to C-terminal phosphorylation; this allows β-arrestin interaction.

Kappa opioid receptor agonists were identified to differentially trigger G protein or β-arrestin coupling (Mores et al., 2019; Faouzi et al., 2020); this capacity, i.e. ligand-dependent activation of some but not other specific transducer of a given GPCR, is called biased agonism (Kenakin, 2017). Very intriguingly, efforts have been made to model biased agonists activity and opioid receptors domain binding: G protein bias involves transmembrane domain V and extracellular loop II, while β-arrestin bias transmembrane domains II and III (Uprety et al., 2021). Moreover, several ligands were studied for their coupling preferences towards the different Gi/o (Olsen et al., 2020). The reason for this focus on the different transductional fingerprints of the kappa opioid receptor lies in the possibility to discriminate its *in vivo* effects i.e. therapeutic vs side-effects. The kappa opioid receptor is a key player in pain and mood modulation. Kappa opioid receptor agonists display a potent antinociceptive effect; however, their clinical use has been strongly limited because dysphoria, hallucinations, and dissociation accompany therapeutic effects. For the kappa opioid receptor, G protein engagement over β-arrestins interaction has been proposed as the preferred profile (Bruchas and Chavkin, 2010). Importantly, therapeutic advantage by kappa opioid receptor activation might be obtained through the development of 1) G protein-biased, 2) peripherally restricted, and 3) mixed opioid agonists (Paton et al., 2020). Intriguingly, nalfurafine is marketed (in Japan) as an antipruritic drug. It is a selective kappa opioid agonist (Inui, 2015), reportedly biased towards G protein (Mores et al., 2019), particularly at human compared to rodent receptors (Schattauer et al., 2017), and it is currently the sole marketed selective kappa opioid receptor agonist. Captivating insights come from novel omics approaches are modifying the scenario of GPCR biased agonism by studying several (virtually all) possible transduction mechanism at a time. Such studies linked the mammalian Target of Rapamycin pathway to the aversive behavior of kappa opioid receptor agonists (Liu et al., 2019).

Blocking the kappa opioid receptor is recommended to treat depressive, anxiety, and substance use disorders (Lutz and Kieffer, 2013; Carlezon and Krystal, 2016). GPCRs residence time (reciprocal of the dissociation rate constant) has been described as good predictor of *in vivo* efficacy (Strasser et al., 2017; van der Velden et al., 2020). However, as stated by Page and co-workers the peculiarly slow pharmacodynamic of kappa opioid receptor antagonists complicate their early phase clinical investigation; therefore, the development of shorter-acting antagonists is needed and might provide insight into whether these drugs are efficacious as predicted in preclinical studies (Carroll and Carlezon, 2013; Page et al., 2019).

In this study we deployed multiple assays to study the pharmacology of standard kappa opioid receptor ligands with differing pharmacological properties then to characterize and compare two novel dynorphin A (Dyn A) derivatives: PWT2-Dyn A and Dyn A-palmitic. PWT2-Dyn A is a tetrabranched derivative obtained by jointing four molecules of [Cys^18^]Dyn A to a PWT2 core. Dyn A-palmitic was obtained by reaction of [Cys^18^]Dyn A with a palmitoyl functionalized maleimide moiety. Specifically, we report data using calcium mobilization, dynamic mass redistribution (DMR), and bioluminescence resonance energy transfer (BRET) receptor-transducer interaction assays.

## Methods

### Drugs

Peptides Dyn A, [D-Pro^10^]Dyn(1-11)-NH_2_ ([D-Pro^10^]) and [Dmt^1^,Tic^2^]Dyn(1-11)-NH_2_ ([Dmt^1^,Tic^2^]) were synthesized in line with solid phase peptide synthesis methodology previously reported (Gairin et al., 1986; Guerrini et al., 1998). The homo tetrameric PWT2-Dyn A was obtained grafting four molecules of [Cys^18^]Dyn A with the PWT2 core in a classical thiol-Michael reaction using experimental conditions previously optimized for the synthesis of nociceptin/orphanin FQ tetra branched derivatives (Guerrini et al., 2014). Similarly, the Dyn A analogue, Dyn A-palmitic was synthesized reacting in solution [Cys^18^]Dyn A with a palmitoyl functionalized maleimide moiety (Pacifico et al., 2020).

The non-peptide molecules U-69,593, Nor-Binaltorphimine (Nor-BNI), and GDP were from Tocris bioscience (Bristol, United Kingdom). All tissues culture media and supplements were from Invitrogen (Paisley, United Kingdom). Reagents used were from Sigma Chemical Co. (Poole, United Kingdom) and were of the highest purity available.

Concentrated solutions of ligands were made in ultrapure water (1 mM, peptides; 10 mM, GDP) or dimethyl sulfoxide (10 mM) and kept at - 20°C until use.

### Calcium Mobilization Assay

Chinese Hamster Ovary (CHO) cells stably co-expressing the human kappa or mu opioid receptors and the Gα_qi5_ protein or the human delta and the Gα_qG66Di5_ protein were used in this assay. Cells were generated as described by Camarda and co-workers (Camarda and Calo, 2013). They were cultured in DMEM/F-12 (1:1) medium supplemented with 10% FBS, 2 mM l-glutamine, 200 mg/ml G418, 100 IU/ml penicillin, 100 IU/ml streptomycin, and 1 μg/ml fungizone. In the assays, cells were maintained at 37°C in a humidified atmosphere with 5% CO_2_. After reaching confluence, cells were detached by trypsinization, and 50,000 cells/well were seeded into 96-well black, clear-bottom plates 24 h before the test. At the assay time, cells were pre-incubated for 30 min at 37°C protected from light with a loading solution consisting of HBSS supplemented with 2.5 mM probenecid, 3 μM Fluo-4 AM, and 0.01% pluronic acid. The loading solution was subsequently discarded, and 100 μL/well of assay buffer consisting of HBSS with 20 mM HEPES, 2.5 mM probenecid, and 500 µM Brilliant Black (Sigma-Aldrich, St. Louis, United States) was dispensed and incubated for an additional 10 min under the same conditions. Serial dilutions of ligands were prepared in HBSS buffer with 20 mM HEPES and 0.02% of bovine serum albumin (BSA) to minimize ligands’ stickiness to plasticware. The automated microplate reader FlexStation II (Molecular Device, Union City, CA 94587, United States) was employed to detect changes in fluorescence intensity. Experiments were carried out at 37°C. Automated additions were carried out in a volume of 50 μL/well. In antagonism experiments, ligands tested as antagonists were administered 24 min before adding the agonist. Three cycles of mixing were performed immediately after injection to ensure proper drug diffusion into the well. The effects of all compounds were expressed as the maximum change in percentage over the baseline fluorescence measured in samples treated with vehicle.

### Dynamic Mass Redistribution

DMR experiments were conducted as previously described (Malfacini et al., 2018). Chinese Hamster Ovary (CHO) cells stably expressing the human kappa receptor were kindly provided by L Toll (Torrey Pines Institute for Molecular Studies, Port St. Lucie, United States). Cells were cultured in Dulbecco’s Modified Eagle Medium: Nutrient Mixture F-12 (DMEM/F12) supplemented with 10% (v/v) Fetal Calf Serum (FCS), 100 U/ml penicillin, 100 μg/ml streptomycin, 2 mM l-glutamine, 15 mM HEPES. The medium was supplemented with 400 μg/ml G418 to maintain expression. Cells were seeded at a density of 45,000 cells/well in 120 μL into fibronectin-coated EnspireTM-LC 96-wells plates and cultured for 20 h to form a confluent monolayer. On the day of the experiment, cells were washed twice and maintained with assay buffer: HBSS with 20 mM HEPES, 0.01% BSA fraction V; for 90 min before the experiment. DMR was monitored in real-time with a temporal resolution of 44 s throughout the assay. Experiments were performed at 37°C, using an EnSight Multimode Plate Reader (PerkinElmer). A 5 min baseline was first established, followed by adding compounds manually in a volume of 40 μL and recording compound-triggered DMR signal for 60 min. In antagonism experiments, ligands tested as antagonists were added 30 min before agonist administration. Maximum picometers (pm) modification (peak) were used to determine agonist response after baseline normalization.

### Bioluminescence Resonance Energy Transfer (BRET) Receptor-Transducer Interaction Assay

*In vitro* pharmacological effects of ligands at kappa opioid receptors were assessed by evaluating receptor interaction with G-protein and β-arrestin 2 with a BRET assay previously set up and validated for delta and mu (Molinari et al., 2010) and NOP (Malfacini et al., 2015) receptors.

SH-SY5Y human neuroblastoma cells permanently co-expressing the different pairs of fusion proteins were prepared using the pantropic retroviral expression system by Clontech as described previously (Molinari et al., 2010). Specifically, human kappa-RLuc fusion protein was made by linking the C-terminal of the receptor sequence without its stop codon, to the N-terminal of RLuc through a 13-mer linker peptide (RTEEQKLISEEDL) and cloned into the retroviral expression vector pQIXN (Clontech). The construction of the plasmids encoding the bovine Gβ1 and the human β-Arrestin 2 N-terminal-tagged with RGFP (Prolume, Pinetop, United States) were previously detailed (Molinari et al., 2010).

SH-SY5Y cells stably co-expressing the fusoproteins kappa-RLuc and Gβ1-RGFP or kappa-RLuc and β-arrestin 2-RGFP were grown in DMEM/F12 (1:1) medium supplemented with 10% FBS, 2 mM l-Glutamine, 100 μg/ml hygromycin B, 400 μg/ml G418, 100 units/ml penicillin G, 100 μg/ml streptomycin and 1 μg/ml Fungizone at 37°C in a humidified atmosphere with of 5% CO_2_. Enriched plasma membrane samples from kappa-RLuc/Gβ1-RGFP expressing cells for receptor-G protein interaction assay were prepared by differential centrifugation and stored at −80°C until use as previously described. Total protein concentration in membrane preparations was determined by colorimetric method with Quantum Protein-BCA kit (EuroClone, Pero (MI), IT) using the multiplate reader Victor Nivo (PerkinElmer, Walthman, MA, United States). BRET assays were performed in white opaque 96 wells microplates (PerkinElmer, Walthman, MA, United States) at room temperature. In kappa opioid receptor-G protein interaction experiments, cell membranes were thawed and resuspended in PBS supplemented with 0.02% BSA, and an amount of 5 μg of total protein was dispensed in each 96 well. In kappa opioid receptor/β-arrestin 2 recruitment experiments 100,000 cells/well were seeded 24 h prior to the BRET test. On the day of the experiment, the complete medium was discarded, and cells were washed with PBS supplemented with 0.5 mM MgCl_2_, 0.9 mM CaCl_2_. Cells were subsequently incubated with 2 μM Prolume Purple Coelenterazine (NanoLight Technology, White Mountain, AZ; United States) for 10 min before bioluminescence reading. Agonists were added and incubated for 5 min (or 30 min where indicated) before microplate reading. In antagonism experiments, antagonists were dispensed into wells 15 min before coelenterazine incubation, Dyn A was subsequently added, and luminescence was acquired. Counts per second (CPS) were detected by Victor luminometer, emissions were selected using 405(10) and 510(30) bandpass filters for Rluc and RGFP, respectively. Acquired data were computed as BRET ratios calculated as follow:

$${\left(\frac{{\text{RGFP}}_{\text{CPS}}}{{\text{RLuc}}_{\text{CPS}}}\right)}_{\text{ligand}}-\;{\left(\frac{{\text{RGFP}}_{\text{CPS}}}{{\text{RLuc}}_{\text{CPS}}}\right)}_{\text{vehicle}}$$

Agonists effects were expressed as a fraction of the maximal effect induced by Dynorphin A following blank (vehicle) subtraction.

RLuc interference experiments were performed on SH-SY5Y cell membranes prepared and quantified as described above. Because several ligands were described to directly interact with RLuc or generate unspecific luminescence artifacts (Auld et al., 2008), the amplitude of ligand-RLuc light alteration was quantified. Cells used expressed the kappa-RLuc and the β-Arrestin 2-RGFP fusoproteins. Following the membrane extraction routine, cell membranes did lose the soluble protein β-Arrestin 2-RGFP but not the kappa-RLuc allowing for precise quantification of direct RLuc light emission alteration. High concentrations of ligands were tested with membranes in the presence of CNTZ, and results were expressed in relation to vehicle/buffer set at 100% (Supplementary Figure S1). 15% alteration of vehicle/buffer RLuc-CNTZ emission was considered as the threshold for excluding a given compound’s concentration from the concentration-response curves in following pharmacological experiments.

### Data Analysis and Terminology

The pharmacological terminology is consistent with the International Union of Basic and Clinical Pharmacology (IUPHAR) recommendations (Neubig et al., 2003). Concentration-response curves to agonists were fitted to the four-parameter logistic nonlinear regression model as follows:

$$\text{Effect}=\text{Basal}+\;\frac{\text{Emax}-\text{Basal}}{1+10^{\;\left({\text{LogEC}}_{50}-\text{Log}\left[\text{ligand}\right]\right)\;\times \text{ HillSlope}}}$$

Curve fitting was performed using PRISM 6.0 (GraphPad Software Inc., San Diego, CA).

In antagonism experiments, concentration-response curves to Dyn A in the absence and in the presence of increasing concentrations of antagonists (Schild analysis) were performed and antagonist potency derived as pA_2_ intercept to the *y*-axis of the linear regression in the Schild plot. If unsurmountable antagonist behavior was detected, the pK_B_ value was derived as follows: a double-reciprocal plot of equi-effective concentrations of agonist (A) in the absence (1/A) and presence (1/A’) of the antagonist (B) was constructed and pK_B_ was derived from the equation pK_B_ = log {(slope-1)/[B]} (Kenakin, 2009).

To quantify the differences in agonist efficacies for G protein and arrestin interactions, bias factors were calculated by choosing the endogenous kappa opioid ligand Dyn A as standard unbiased ligand. For this analysis, the E_max_ and EC_50_ of the agonist were derived using a 3-parameter logistic model with slope values equal to unity. Although several agonist curves displayed slope values different from 1, refitting the curves with the parameter fixed to unity did not produce a statistically significant reduction of the goodness of fit. Under such conditions, the relative ratio (Emax/EC_50_)_lig_/(Emax/EC_50_)_Dyn A_ is equivalent to the relative (τ/K)_lig_/(τ/K)_Dyn A_ ratio as defined by the operational model (Black and Leff, 1983; Griffin et al., 2007). Taking ratios of these values between G protein and arrestin can cancel the common K and yield the ratio of ligand intrinsic efficacy across the two transduction proteins. Thus, the following formula was used for calculating agonist bias factors in log_10_ units:

bias factor= log[(EmaxEC50)lig(EmaxEC50)Dyn A]G prot.−log[(EmaxEC50)lig(EmaxEC50)Dyn A]β−arr.

Bias factors were considered significantly different from the reference ligand when a ligand’s CL_95%_ did not include zero. The pharmacological terminology and computations related to biased agonism were consistent with IUPHAR recommendations (Kolb et al., 2022).

Data are expressed as mean ± sem of n experiments and were analyzed statistically using one-way analysis of variance followed by Dunnett’s test for multiple comparisons. Potency values and bias factors are expressed as mean (CL_95%_). *p* values <0.05 were considered statistically significant.

## Results

### Calcium Mobilization Assay

**FIGURE 1:**
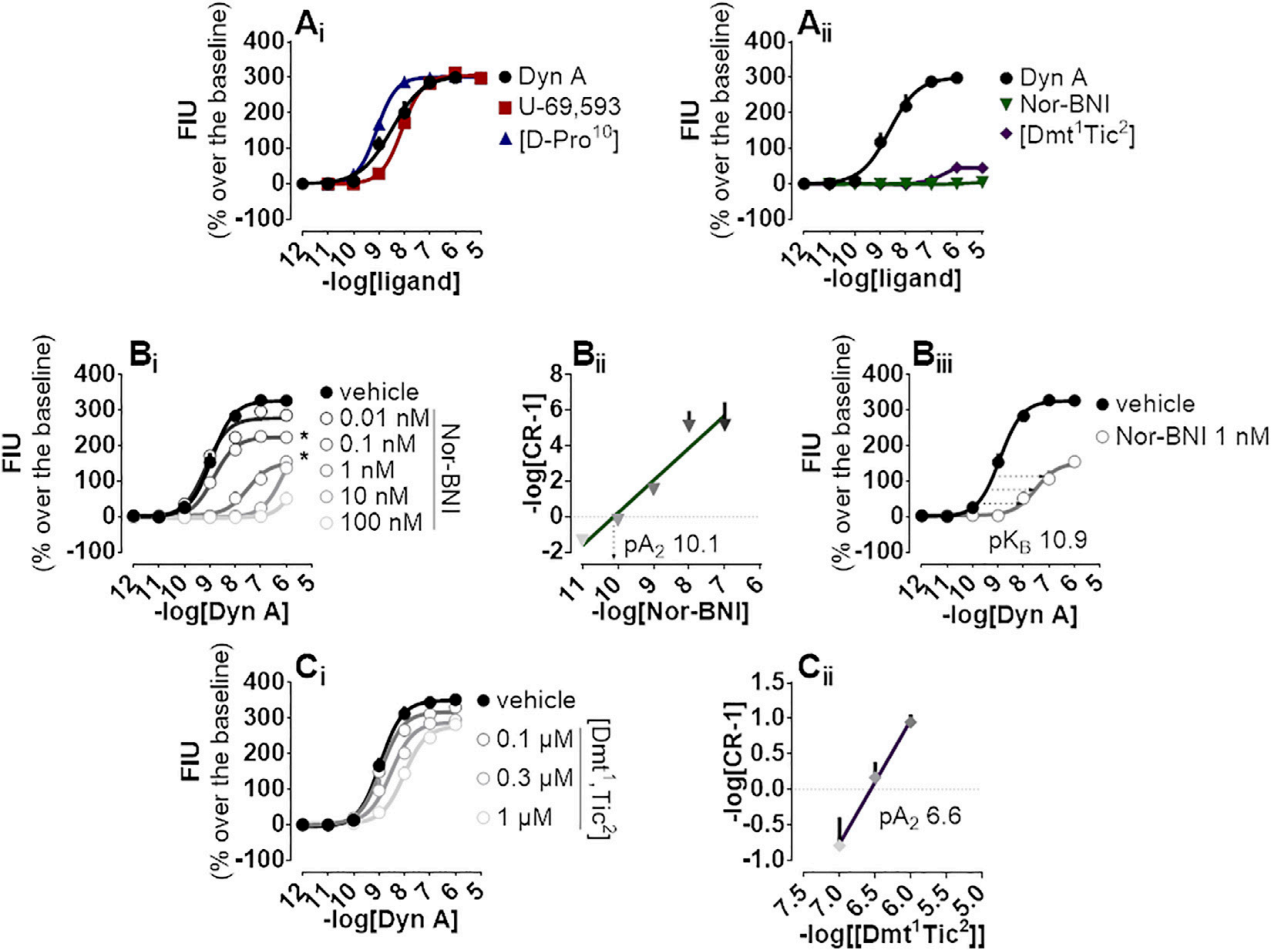
Calcium mobilization experiments carried out in CHO cells stably expressing the human kappa opioid receptor and the Gα_qi5_ chimeric G protein **(A_i_)** Dyn A, U-69,593, and [D-Pro^10^], and **(A_ii_)** Nor-BNI and [Dmt^1^,Tic^2^] concentration-response curves **(B_**i**_)** Concentration-response curves to Dyn A in the absence and in the presence of increasing (10 p.m.—100 nM) concentrations of Nor-BNI, corresponding **(B_**ii**_)** Schild analysis and **(B_**iii**_)** computation of antagonist potency for non-competitive antagonists **(C_**i**_)** Concentration-response curves to Dyn A in the presence of increasing concentrations (0.1—1 µM) of [Dmt^1^,Tic^2^] with corresponding **(C_**i**_)** Schild analysis. Data are mean +sem of three to six independent experiments performed in duplicate and Emax were analyzed statistically using one-way analysis of variance followed by Dunnett’s test for multiple comparisons. *p* values <0.05 were considered statistically significant and labelled with *.

The endogenous peptide Dyn A, the synthetic peptide [D-Pro^10^], and the non-peptide arylacetamide U-69,593 were chosen as standard kappa opioid agonists and tested on CHO cells stably co-expressing the kappa opioid receptor together with signaling altered chimeric G protein (Gα_qi5_) enabling calcium mobilization assaying. In this system, all compounds evoked high maximal effects (Emax, approx. 300% fluorescence intensity units (FIU) over the baseline) and displayed high potency ranging from 9.08 for [D-Pro^10^] to 8.09 for U-69,593 (Figure 1Ai). Conversely, peptide [Dmt^1^,Tic^2^] and non-peptide Nor-BNI antagonists did not show high stimulation of calcium flux, with [Dmt^1^,Tic^2^] increasing the calcium levels to 50% over the baseline only at micromolar concentrations and Nor-BNI being completely inactive (Figure 1Aii). Same antagonists were investigated for their capacity to block kappa opioid receptor activation in Schild protocol experiments where the effects of increasing concentrations of Dyn A were challenged with different concentrations of the two molecules. Nor-BNI produced a dextral shift in the Dyn A concentration-response curve with a progressive depression of maximal effects (Figure 1Bi); for this reason, the estimation of Nor-BNI potency as shown in the Schild plot (Figure 1Bii) was further corroborated by the approach shown in Figure 1Biii. Both computations returned very high potency values (pA_2_ 10.1 and pK_B_ 10.9, respectively). [Dmt^1^,Tic^2^] produced a rightward shift in Dyn A concentration-response curve without modifying the agonist maximal effects (Figure 1Ci); a pA_2_ value of 6.6 was derived from these experiments (Figure 1Cii).

### DMR Assay

**FIGURE 2:**
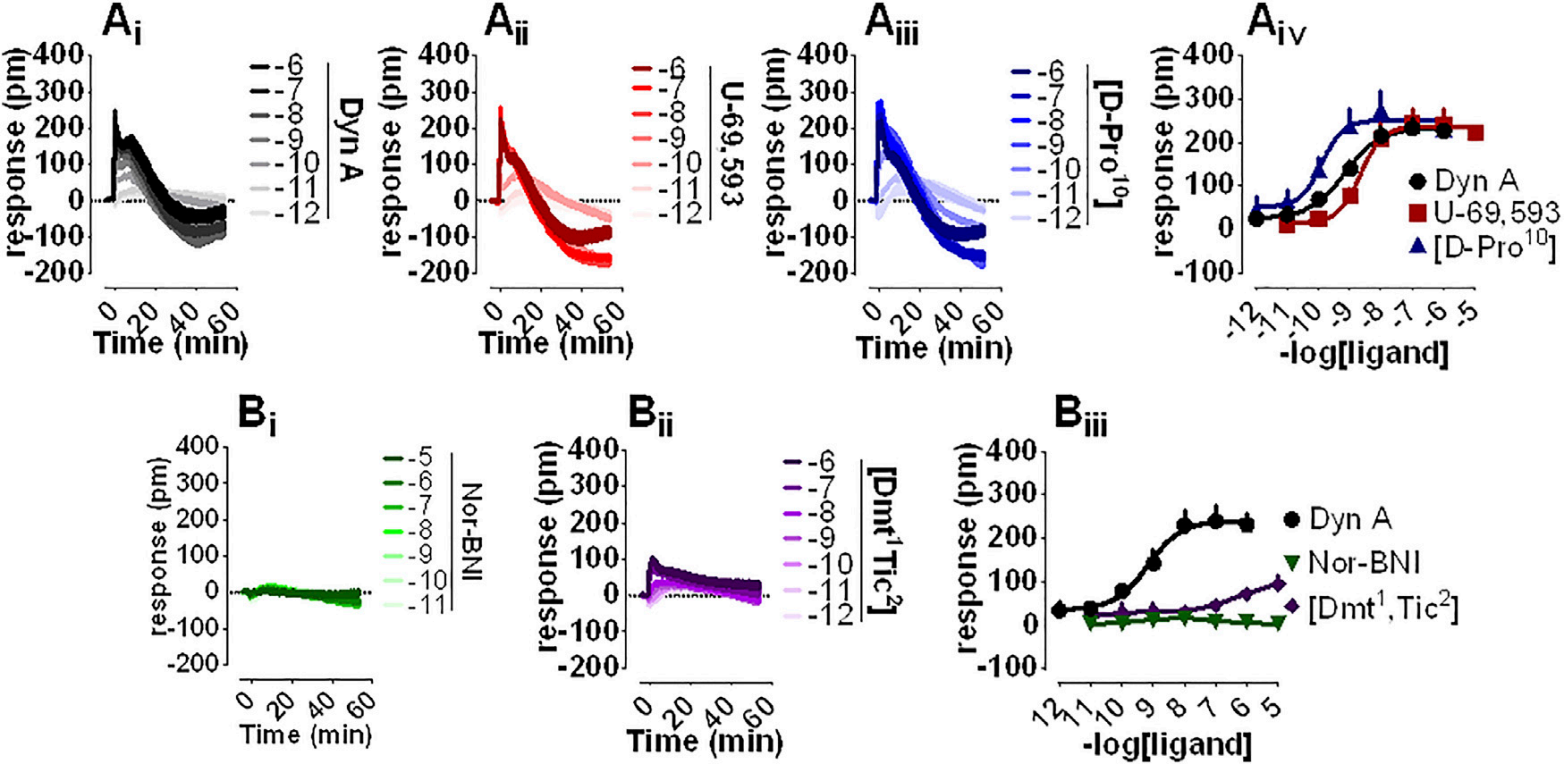
DMR experiments in CHO cells stably expressing the human kappa opioid receptor **(A_**i-iii**_)** Averaged kinetics of increasing concentrations of Dyn A, U-69,593, and [D-Pro^10^], and **(A_**iv**_)** corresponding concentration-response curves **(B_**i-ii**_)** Averaged effects of Nor-BNI and [Dmt^1^,Tic^2^], and **(B_**iii**_)** corresponding concentration-response curves. Data represented are mean +sem of four to seven independent experiments performed in duplicate and Emax were analyzed statistically using one-way analysis of variance followed by Dunnett’s test for multiple comparisons.

DMR is a noninvasive, unbiased, label-free approach that was employed to assess the pharmacological profile of human kappa opioid receptors expressed in CHO cells. Dyn A (Figure 2Ai), U-69,593 (Figure 2Aii), and [D-Pro^10^] (Figure 2Aiii) elicited a concentration-dependent positive DMR signal, with Emax spanning from 234 to 251 pm (pm) for Dyn A and [D-Pro^10^], respectively. Potencies for Dyn A, U-69,593, and [D-Pro^10^] were 9.15, 8.60, and 9.84 (Figure 2Aiv). Nor-BNI (Figure 2Bi) did not modify DMR baseline while [Dmt^1^,Tic^2^] (Figure 2Bii) slightly stimulated the DMR response only at micromolar concentrations (Figure 2Biii).

**FIGURE 3:**
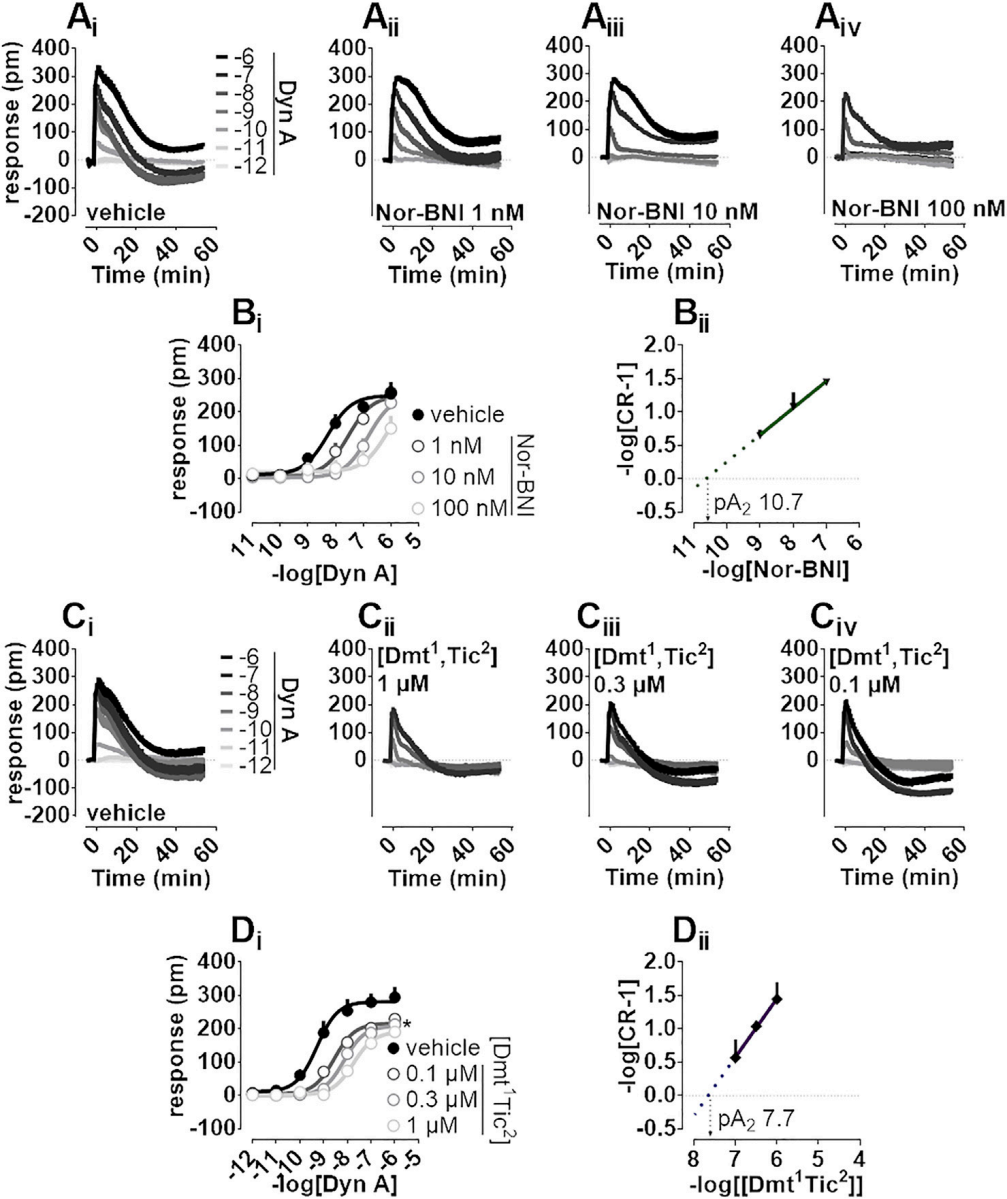
DMR experiments in CHO cells stably expressing the human kappa opioid receptor **(A_**i-iv**_)** Averaged kinetic traces of increasing (1 p.m.—1 µM) concentrations of Dyn A in the absence and in the presence of different (1–100 nM) concentrations of Nor-BNI, corresponding **(B_**i**_)** concentration-response curves and **(B_**ii**_)** Schild analysis **(C_**i-iv**_)** Averaged kinetic traces of increasing Dyn A concentrations in the absence and in the presence of different (0.1–1 µM) concentrations of [Dmt^1^,Tic^2^] with corresponding **(D_**i**_)** concentration-response curves and **(D_**ii**_)** Schild analysis. Data represented are mean +sem of five to seven independent experiments performed in duplicate and Emax were analyzed statistically using one-way analysis of variance followed by Dunnett’s test for multiple comparisons. *p* values <0.05 were considered statistically significant and labelled with *.

Nor-BNI and [Dmt^1^,Tic^2^] antagonist properties were evaluated by Schild analysis. Comparison of representative Dyn A concentration-effect curve alone (Figure 3Ai) to that of Dyn A in the presence of single concentrations of Nor-BNI (1—100 nM, Figure 3Aii-iv) are shown. Nor-BNI concentration-dependently shifted the sigmoidal curves of Dyn A to the right without significantly affecting Emax (Figure 3Bi). pA_2_ from Schild plot extrapolation was 10.7 (Figure 3Bii). Dyn A alone (Figure 3Ci) and following preincubation with [Dmt^1^,Tic^2^] at different single concentrations (0.1—1 μM, Figure 3Cii-iv) are shown. DMR response to Dyn A was slightly depressed by all concentrations of antagonist used (Figure 3Di), likely because the weak stimulatory effect of [Dmt^1^,Tic^2^] (as in Figure 2Bii, Biii) is subtracted from the baseline by the instrument software. In Schild analysis of [Dmt^1^,Tic^2^] against Dyn Aa pA_2_ of 7.7 was calculated.

### BRET Assay

**FIGURE 4:**
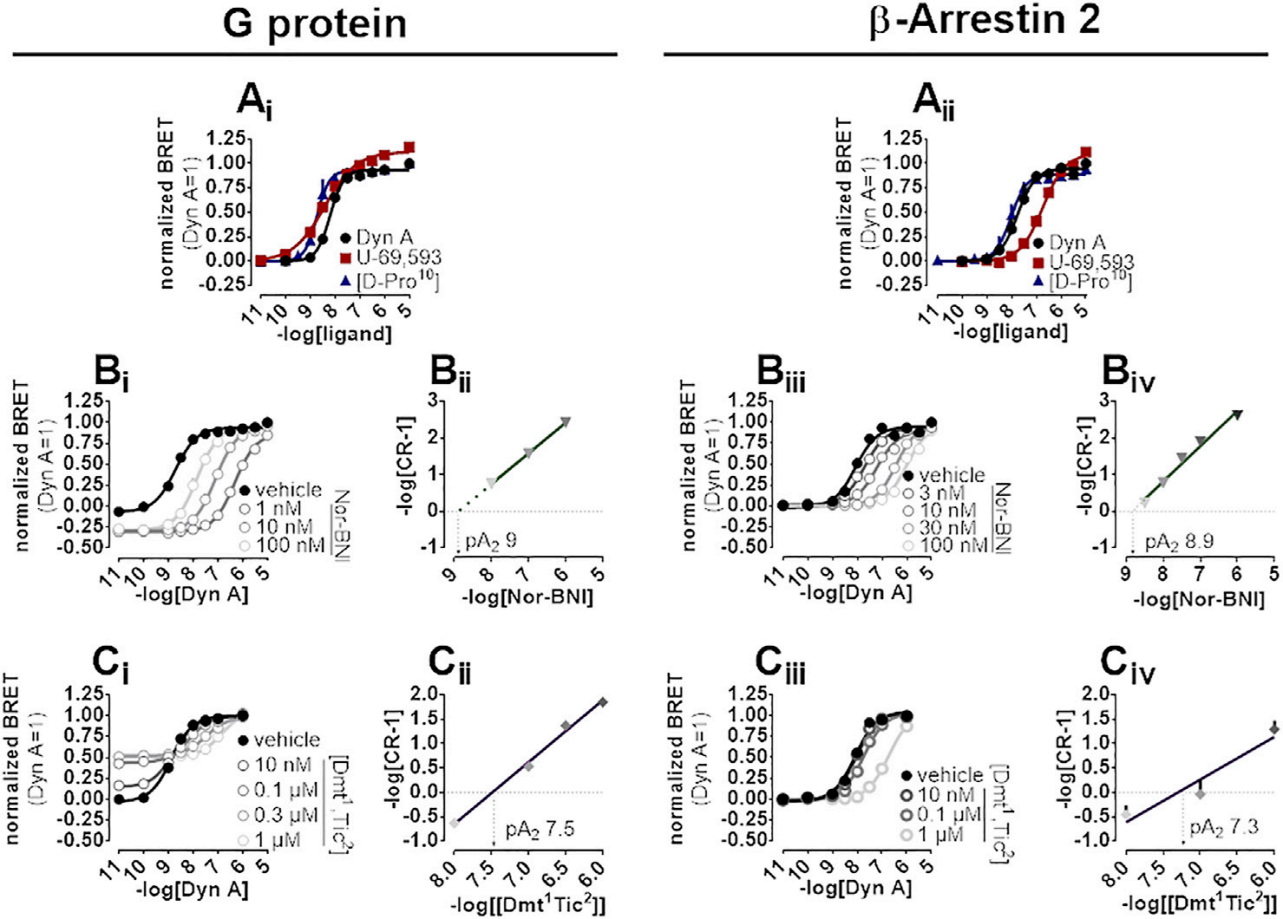
BRET experiments in SH-SY5Y cells stably expressing the human kappa opioid-RLuc tagged receptor together with either the Gβ_1_-RGFP or the β-Arrestin 2-RGFP fusoprotein. Concentration-response curves to Dyn A, U-69,593, and [D-Pro^10^] on the stimulation of kappa-G protein **(A_**i**_)** and kappa-β-Arrestin 2 **(A_**ii**_)** interaction. Concentration-response curves to Dyn A in the absence and in the presence of increasing concentrations of Nor-BNI on the stimulation of kappa-G protein **(B_**i**_)** and kappa-β-Arrestin 2 **(B_**iii**_)** interactions with corresponding Schild plots **(B**_**ii**_ and **B**_**iv**_, respectively**)**. Concentration-response curves to Dyn A in the absence and in the presence of increasing concentrations of [Dmt^1^,Tic^2^] on the stimulation of kappa-G protein **(C_**i**_)** and kappa-β-Arrestin 2 **(C_**iii**_)** interaction, with corresponding Schild analysis **(C**_**ii**_ and **C**_**iv**_, respectively**)**. Data represented are mean +sem of three to five independent experiments performed in duplicate.

A BRET approach enabling the measurement of kappa opioid receptor to G protein (Gβ_1_) or to β-Arrestin 2 interaction was employed to test the activity of kappa opioid receptor ligands. In the kappa-G protein interaction assay, Dyn A, U-69,593, and [D-Pro^10^] displayed similar maximal effects and potency (pEC_50_) of 8.21, 8.52, and 8.36, respectively (Figure 4Ai). The interaction of the β-Arrestin 2 to the kappa opioid receptor was also promoted by Dyn A, U-69,593, and [D-Pro^10^] with similar high maximal effects, and pEC_50_ values of 7.74, 6.72, and 8.07, respectively (Figure 4Aii). The computation of bias factors (towards G protein) for U-69,593 and [D-Pro^10^] returned values of 1.22 and 0.29, respectively (Supplementary Figure S2).

In kappa-G protein interaction experiments, Nor-BNI (1–100 nM) shifted the concentration-effect curves to Dyn A to the right without altering maximal effects (Figure 4Bi). A pA_2_ of nine was extrapolated from the Schild plot (Figure 4Bii). Because Nor-BNI appeared to depress the baseline of kappa-G protein interaction, and, since this assay is performed on cell membranes, GDP can be applied to investigate receptor constitutive activity (see (Vezzi et al., 2013)) for similar experiments at delta and mu opioid receptors), Nor-BNI activity was compared to that of GDP at 5 and 30 min. Experiments (Supplementary Figure S3, Supplementary Table S1) showed how Nor-BNI depression of baseline, detected also with GDP, was visible only with prolonged incubation time. Nor-BNI, in addition, generated a robust shift to the right of Dyn A response in kappa-β-Arrestin 2 interaction, with no modification of maximal effects (Figure 4Biii). pA_2_ value obtained from the Schild plot was of 8.9 (Figure 4Biv). In kappa-G protein interaction experiments [Dmt^1^,Tic^2^] concentration-dependently increased baseline (Supplementary Figure S3) and elicited a dextral displacement of the concentration-response curve to Dyn A without altering maximal effects (Figure 4Ci). Schild analysis of these results yielded a pA_2_ of 7.5 (Figure 4Cii). In kappa-β-Arrestin 2 interaction experiments, [Dmt^1^,Tic^2^] did not show any residual agonist activity but produced a dextral displacement of the Dyn A concentration-response curve without alteration of the agonist maximal effect (Figure 4Ciii); a pA_2_ value of 7.3 was derived from these experiments (Figure 4Civ).

### PWT2-Dyn A

**FIGURE 5:**
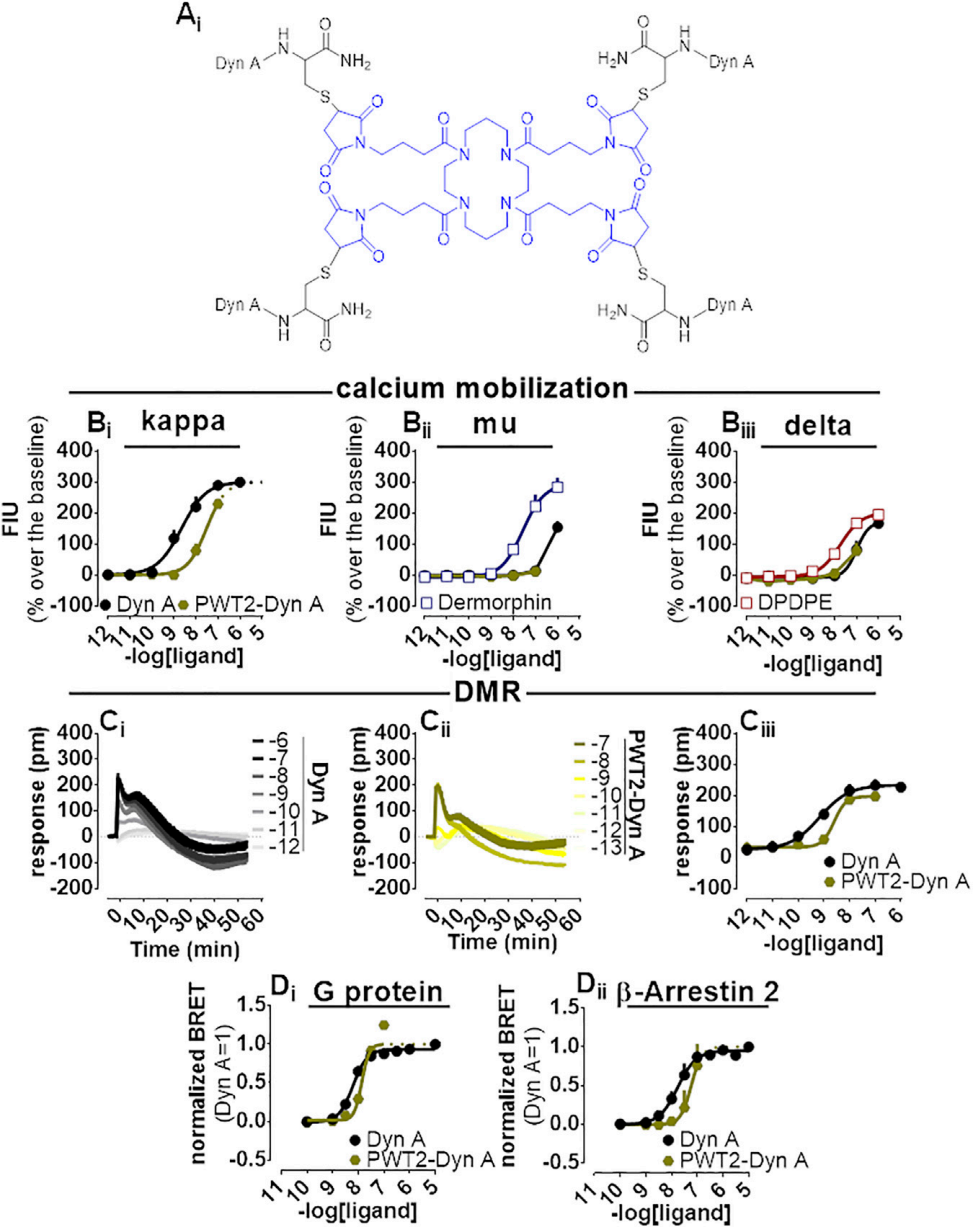
Pharmacological characterization of PWT2-Dyn A. Molecular structure **(A_**i**_)**. Calcium mobilization experiments in CHO stably expressing the human kappa **(B)**, mu **(Bii)i** and delta **(Bii)** opioid receptor and chimeric G proteins, concentration-response curves to Dyn A and PWT2-Dyn A. Averaged DMR traces obtained in CHO cells stably expressing the human kappa opioid for increasing concentration of Dyn A **(C_**i**_)** and PWT2-Dyn A **(C_**ii**_)**, with corresponding concentration-response curves **(C_**iii**_)**. Concentration response curves to Dyn A and PWT2-Dyn A performed with a BRET assay on SH-SY5Y cells stably expressing the human kappa opioid-RLuc tagged receptor together with either the Gβ_1_-RGFP **(D_**i**_)** or the β-Arrestin 2-RGFP fusoprotein **(D_ii_)**. Data are mean +sem of three to six independent experiments performed in duplicate.

PWT2-Dyn A is a tetrabranched derivative of Dyn A (Figure 5Ai) whose high-performance liquid chromatography and mass spectrometric analysis are shown in Supplementary Figure S4. PWT2-Dyn A was capable of evoking concentration-dependent increase of calcium mobilization in CHO cells stably expressing kappa opioid receptors and chimeric G proteins. The effects of PWT2-Dyn A were assayed up to 100 nM, and maximal effects were constrained to be the same as Dyn A (299% over the baseline) with a potency (pEC_50_) derived for PWT2-Dyn A of 7.51 (Dyn A 8.53) (Figure 5Bi). CHO cells stably expressing either the mu opioid receptor and the Gα_qi5_ chimeric G protein or the delta opioid receptor and the Gα_qG66Di5_ chimeric G protein were employed to study the selectivity of action of PWT2-Dyn A over the mu and the delta opioid receptors. Dermorphin displayed high maximal effects (294% over the baseline) and moderate potency (pEC_50_ 7.57) in cells expressing the mu opioid receptor. Dyn A produced a stimulation (154% over the baseline) only at the highest concentration tested. PWT2-Dyn A was inactive (Figure 5Bii). DPDPE was tested as a standard delta opioid agonist, its maximal effects (202% over the baseline) and potency (pEC_50_ 7.74) were found high, Dyn A also displayed high maximal effects (171% over the baseline) albeit with lower (pEC_50_ 7.00) potency and PWT2-Dyn A exerted a slight (77% over the baseline) stimulatory effect only at the highest concentration tested (Figure 5Biii).

When Dyn A and PWT2-Dyn A activities were compared in the DMR assay with CHO cells stably expressing the kappa opioid receptor (Figure 5Ci-iii), PWT2-Dyn A mimicked the stimulatory effect exerted by the natural peptide Dyn A with similar maximal effects and 4-fold lower potency (pEC_50_ 8.51).

In the BRET assay, only concentrations of PWT2-Dyn A up to 10 nM could be tested because of unclear alteration of the RLuc emitted light (Supplementary Figure S1). Nevertheless, 10 nM PWT2-Dyn A reached the maximal effects of Dyn A in the kappa-G protein (Figure 5Di) and kappa-β-Arrestin 2 (Figure 5Dii) interaction assays, with derived potency values 3-fold lower than Dyn A in both assays.

### Dyn A-Palmitic

**FIGURE 6:**
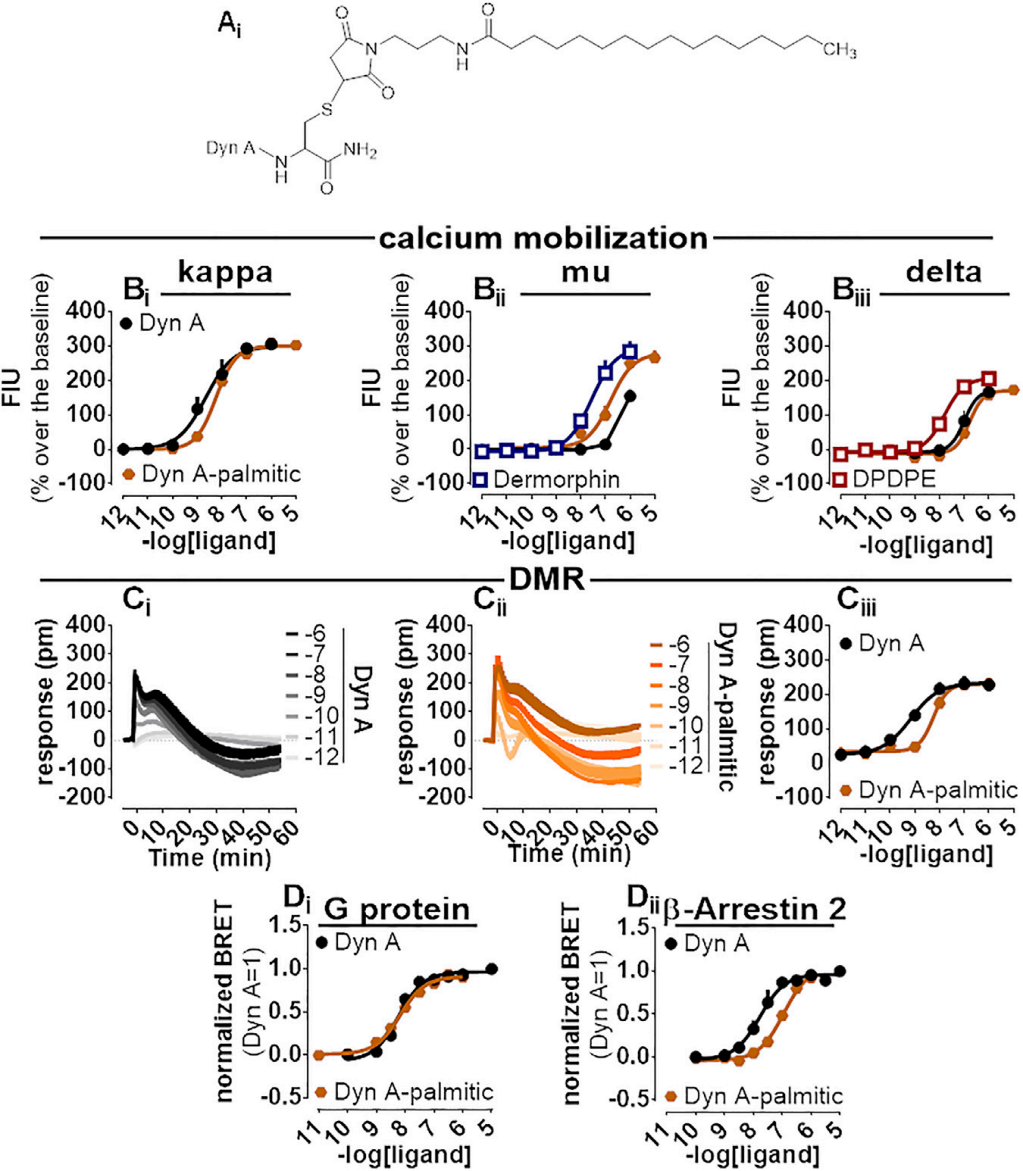
Pharmacological characterization of Dyn A-palmitic. Molecular structure **(A_**i**_)**. Calcium mobilization experiments in CHO cells stably expressing the human kappa **(B_**i**_)**, mu **(B_**ii**_)**, and delta **(B_**iii**_)** opioid receptors and chimeric G proteins. Concentration-response curves to Dyn A and Dyn A-palmitic. Averaged DMR traces obtained in CHO cells stably expressing the human kappa opioid for increasing concentration of Dyn A **(C_**i**_)** and Dyn A-palmitic **(C_**ii**_)**, with corresponding concentration-response curves **(C_**iii**_)**. Concentration response curves to Dyn A and Dyn A-palmitic performed with a BRET assay on SH-SY5Y cells stably expressing the human kappa opioid-RLuc tagged receptor together with either the Gβ_1_-RGFP **(D_**i**_)** or the β-Arrestin 2-RGFP fusoprotein **(D_**ii**_)**. Data are mean +sem of three to six independent experiments performed in duplicate.

A palmitoylated derivative of Dyn A, Dyn A-palmitic (Figure 6Ai), was examined. Dyn A-palmitic high-performance liquid chromatography and mass spectrometric analysis are shown in Supplementary Figure S5. In calcium mobilization studies performed with kappa receptor-expressing cells, Dyn A-palmitic displayed high maximal effects similar to that of Dyn A with only 2-fold loss of potency (Figure 6Bi). The selectivity of Dyn A-palmitic over the mu and the delta opioid receptors was assayed as for PWT2-Dyn A. Dyn A-palmitic, mimicked the stimulatory effects of dermorphin, with an approximately 6-fold lower potency (Figure 6Bii). At delta opioid receptor-expressing cells Dyn A-palmitic was 10-fold less potent than DPDPE (Figure 6Biii).

Dyn A-palmitic was tested on the DMR assay together with Dyn A (Figure 6Ci, Cii), both compounds elicited high maximal effects in CHO cells expressing the kappa opioid receptor, with Dyn A-palmitic showing 6-fold lower potency than Dyn A (Figure 6Ciii).

In the BRET assay for kappa-G protein interaction, Dyn A and Dyn A-palmitic concentration-response curves were superimposable (potency ∼6 nM) (Figure 6Di). In kappa-β Arrestin-2 interaction experiments and despite mimicking the stimulatory effects of Dyn A with similar maximal effects, Dyn A-palmitic showed 7-fold lower potency than the natural peptide (Figure 6Dii). A bias factor (towards G protein) of 0.74 was derived from these experiments (Supplementary Figure S2).

All pharmacological parameters with corresponding CL_95%_ or sem are summarized in Table 1. Results obtained in terms of biased agonism are plotted as bias plot and bias factors bar diagrams in Supplementary Figure S2.

**TABLE 1:** Pharmacological parameters and relative CL_95%_ for Dyn A, U-69,593, [D-Pro^10^], Nor-BNI, [Dmt^1^, Tic^2^], PWT2-DynA, and DynA-Palmitic in the indicated assays.

	Calcium Mobilization			DMR			BRET						
							G Protein			β-Arrestin 2			Bias factor^a^ (CL_95%_)
	pEC_50_ (CL_95%_)	α	pA_2_ (CL_95%_)	pEC_50_ (CL_95%_)	α	pA_2_ (CL_95%_)	pEC_50_ (CL_95%_)	Α	pA_2_ (CL_95%_)	pEC_50_ (CL_95%_)	α	pA_2_ (CL_95%_)	
Dyn A	8.53 (7.94–9.12)	1.00	—	9.15 (8.56–9.74)	1.00	—	8.21 (8.12–8.30)	1.00	—	7.74 (7.33–8.15)	1.00	—	0.00
U-69,593	8.09 (7.93–8.26)	1.00	—	8.60 (8.21–8.98)	1.01	—	8.52 (8.27–8.78)	1.17	—	6.72 (6.37–7.06)	1.12	—	1.22 (0.87–1.54)
[D-Pro^10^]	9.08 (8.93–9.23)	1.00	—	9.84 (9.31–10.37)	1.07	—	8.36 (6.54–10.19)	1.00	—	8.07 (7.66–8.47)	0.95	—	0.29 (-0.06–0.64)
Nor-BNI	inactive		10.1^b^ (9.41-11.51)	inactive		10.7 (9.42–11.98)	8.40 (6.46–10.35)	- 0.11	9 (8.65–9.20)	inactive		8.9 (8.50–9.38)	—
[Dmt^1^,Tic^2^]	∼6.9	0.15	6.6 (6.16–7.14)	7.11 (5.61–8.61)	0.47	7.7 (7.11–8.29)	6.95 (6.14–7.75)	0.35	7.5 (5.35–9.65)	inactive		7.3 (6.5–8.1)	—
PWT2-DynA	7.51 (6.91–8.11)	∼1.00	—	8.51 (9.3–10.37)	0.84	—	7.73 (7.41–8.04)	∼1.00	—	6.66 (6.30–7.02)	∼1.00	—	∼0.18^c^
DynA-Palmitic	8.24 (7.91–8.56)	1.01	—	8.38 (8.06–8.70)	0.99	—	8.17 (7.94–8.40)	0.90	—	6.90 (6.76–7.04)	0.92	—	0.74 (0.52–0.96)

α is the intrinsic activity of a ligand: calculated as the maximal effect of a given agonist divided that of Dyn A.

a Bias factors were calculated using the naturally occurring peptide Dyn A as unbiased agonist.

b Nor-BNI, antagonist potency in the calcium mobilization assay was also determined with the double-reciprocal approach as described in the method section and gave a pK_B_, of 10.9 (10.55-11.25).

c Because of interaction of PWT2-Dyn A with RLuc, light, in BRET, experiments Emax was set equal to one and an approximation of bias factor computed (without error estimation).

## Discussion

The kappa opioid receptor controls important biological functions, and its pharmacological modulation has therapeutic potential. For instance, selective kappa receptor antagonists promote robust anxiolytic and antidepressant-like effects and can also be investigated as innovative drugs to treat substance use disorders (Lutz and Kieffer, 2013; Carlezon and Krystal, 2016). Whilst, kappa receptor agonists display analgesic/antinociceptive and antipruritic (e.g. the Japanese marketed nalfurafine (Inui, 2015)) properties, clinical development is limited by important side effects such as dysphoria, sedation, hallucinations, and diuresis. To increase the therapeutic index of kappa receptor agonists, different strategies have been proposed, including G protein-biased agonists, peripherally restricted molecules, and mixed opioid receptor agonists (Paton et al., 2020).

The aim of this study was twofold, establishing a panel of assays to finely study kappa opioid receptor pharmacology and characterize novel ligands: PWT2-Dyn A and Dyn A-palmitic. We, therefore, deployed an array of assays to investigate the pharmacological profile of the human kappa opioid receptor in recombinant cells. The kappa opioid receptor natively couples to Gi/o heterotrimeric G proteins (Paton et al., 2020); therefore, we could measure calcium mobilization (a prototypical Gq signal) only adopting chimeric Gq/i proteins as described before by us (Camarda and Calo, 2013) and others (Zhang et al., 2012). DMR, an integrated, unbiased approach to assess real-time activation of intracellular signaling, was also employed as previously described for different GPCRs (Schroder et al., 2011), including opioid receptors (Morse et al., 2011; Grundmann et al., 2018). Finally, as previously reported for other opioid receptors (Molinari et al., 2010; Vezzi et al., 2013; Malfacini et al., 2015), two BRET approaches for measuring kappa-G protein and kappa-β-Arrestin 2 interaction were used to further investigate the pharmacology of kappa ligands, and eventually estimate their bias factor. Collectively, the pharmacological fingerprint of the kappa opioid receptor, obtained with potent and selective agonists and antagonists, is in line to that reported in the literature (Alexander et al., 2021). Novel ligands, PWT2-Dyn A and Dyn A-palmitic, showed similar pharmacology to that of the parental natural peptide Dyn A although with slight decrease of potencies. In addition, Dyn A-palmitic showed a significant bias towards G protein.

### Agonists Pharmacological Fingerprint 

Collectively, in our assays, Dyn A, U-69,593, and [D-Pro^10^] behaved as full agonists of the human kappa opioid receptor with the following rank order of potency [D-Pro^10^] > Dyn A ≥ U-69,593, which is in line with several studies employing different assays and preparations (cells and isolated tissues), and multiple isoforms of the kappa opioid receptor (human, mouse, and guinea pig) (Gairin et al., 1984; Lahti et al., 1985; Gairin et al., 1988b; Meng et al., 1993; Yasuda et al., 1993; Soderstrom et al., 1997). Dyn A elicited robust activation of the kappa opioid receptor with potency ranging from 1 to 6 nM, except for the kappa-β-arrestin 2 interaction assay with a slightly lower potency of 18 nM. Similar results were obtained in calcium mobilization studies (Camarda and Calo, 2013). For stimulation of [^35^S]GTPγS binding, potency values obtained were approximately one order of magnitude higher (Zhu et al., 1997). U-69,593 potencies ranked from 3 to 8 nM with 191 nM in the kappa-β-arrestin 2 interaction assay, demonstrating a robust bias towards G protein activation. Literature data showed similar potency values in receptor binding assay of 4 nM, and forskolin stimulated inhibition of cAMP of 17 nM (Katsumata et al., 1996). However, G protein-biased behavior of U-69,593 is not corroborated by literature findings. In CHO cells expressing the human kappa receptor, U-69,593 displayed similar potency and efficacy in [^35^S]GTPγS stimulation and β-arrestin 2 recruitment experiments; however, the effects of U-69,593 were not compared to those of Dyn A (Ho et al., 2018). In the study by White and co-workers (White et al., 2014), U-69,593 has been classified as an unbiased kappa agonist, but Salvinorin A rather than Dyn A was used as a reference ligand. Finally, U-69,593 was also reported to be biased for internalization versus [^35^S]GTPγS stimulation (DiMattio et al., 2015). These inconsistencies underline the need of adopting more stringent rules in the design and reporting of biased agonism studies; these rules have been recently made available by IUPHAR (Kolb et al., 2022).

[D-Pro^10^] is a relatively poorly studied, highly potent Dyn A derivative. Developed in 1984 (Arimitsu et al., 1977), non-amidated [D-Pro^10^]Dyn(1-11) displayed a 7-fold higher affinity to the kappa opioid receptor (detected with [^3^H]Bremazocine binding) than Dyn A and showed 62- and 233-fold selectivity over the mu and delta opioid receptors, respectively. This peptide has been used as a probe to visualize kappa-opioid receptor sites in autoradiographic studies (Jomary et al., 1988). Intracerebroventricular administration of [D-Pro^10^]Dyn(1-11) in mice did not show any activity against thermal stimulus (tail-flick), but produced a dose-dependent antinociceptive effect in the acetic acid-induced writhing model of visceral pain, in line with a kappa-driven agonist activity (Gairin et al., 1988a). Our results confirm the high potency and full agonist activity of this peptide as a kappa agonist. In addition, the present findings suggest that the [D-Pro^10^] chemical modification does not affect the unbiased agonist features of the naturally occurring peptide Dyn A.

### Antagonists Pharmacological Fingerprint 

As far as antagonism is concerned, Dyn A concentration-response curve was challenged by increasing concentrations of Nor-BNI and [Dmt^1^,Tic^2^] in all assays adopted. Nor-BNI displayed potency values ranging 0.01–1.26 nM. [Dmt^1^,Tic^2^] potency, was lower and ranged from 20 to 251 nM. The pharmacological activity of Nor-BNI is in line with the literature, its affinity reported is approximately 1 nM (Simonin et al., 1995) and potency 0.1 nM (Zhu et al., 1997) (Birch et al., 1987). Regarding the type of antagonism, because the calcium mobilization assay was the only one showing an unsurmountable type of antagonism for Nor-BNI, we still classify Nor-BNI as a competitive antagonist. Unsurmountable/noncompetitive-like behaviors can be measured when antagonist-receptor complexes are reversible per se but dissociate too slowly that only a fraction of the receptors are accessible to subsequent stimulation by the agonist. In the calcium mobilization assay, the short time in which agonist effects are measured does not allow equilibration to be reached, especially when the antagonist slowly dissociates from the receptor (Charlton and Vauquelin, 2010). In fact, Nor-BNI is a very long-lasting antagonist *in vivo*, displaying an irreversible-like behavior (Jones and Holtzman, 1992) [Dmt^1^,Tic^2^] was developed in 1998 (Guerrini et al., 1998) and showed affinity values of 3.3—4.3 nM, with equal affinity at the mu opioid receptor and 10-fold higher affinity at the delta opioid receptor. Bioassay experiments on isolated tissues gave antagonist potency values of 90 and 8 nM for this peptide in guinea pig ileum and rabbit jejunum, respectively. The present results demonstrated that [Dmt^1^,Tic^2^] is actually a low efficacy (α 0.15–0.3) partial agonist at the human kappa receptor; of note the peptide behaved as a pure antagonist only in the kappa-β-arrestin 2 interaction assay. As expected partial agonist potency of [Dmt^1^,Tic^2^] was close to antagonistic potency values measured in the same assay. Compounds like [Dmt^1^,Tic^2^] characterized by mixed opioid receptor activity and different degrees of efficacy are currently considered of great interest (Paton et al., 2020); clearly further studies are needed to investigate the beneficial versus unwanted effects elicited *in vivo* by this opioid receptor ligand.

### Inverse Agonism

In kappa-G protein interaction experiments, Nor-BNI not only antagonized the effects of Dyn A but produced a concentration-dependent reduction in the baseline, thus behaving as an inverse agonist. Interestingly, in line with the slow kinetics (see before) of this molecule, this effect was evident only with prolonged incubation time. In addition, kappa opioid receptor constitutive activity is confirmed by experiments with GDP that, being capable of inhibiting the activated G protein, can elicit a condition of virtual zero receptor activation (Vezzi et al., 2013). Since the maximal inhibitory effect of Nor-BNI is similar to that of GDP, Nor-BNI should be classified as a full inverse agonist. As expected, the pEC_50_ of Nor-BNI studied as inverse agonist was close to the pA_2_ obtained investigating the molecule as antagonist. The present results corroborate previous findings obtained in [^35^S]GTPγS binding studies performed with HEK cells expressing the kappa receptor both in term of inverse agonist behavior of Nor-BNI and amount of kappa receptor constitutive activity (10–15% of basal values) (Wang et al., 2007). The liability of the kappa receptor to display constitutive activity seems to be similar to that of the mu receptor and much lower to that of the delta opioid receptor (Wang et al., 2007; Vezzi et al., 2013). Indeed, constitutive activity at GPCRs was first described in cells expressing delta receptors (Costa and Herz, 1989). As far as the possible physiological role of kappa receptor constitutive activity is concerned, little information is available in the literature; however, there is evidence suggesting that constitutively active kappa receptors are expressed in the ventral tegmental area (Polter et al., 2017) and in the medial prefrontal cortex (Sirohi and Walker, 2015) where may regulate response to stress in relation to substance use disorders and impulsivity, respectively.

### PWT2-Dyn A and Dyn A-Palmitic

Finally, we assessed how PWT2-Dyn A and Dyn A-palmitic behave in our pharmacological assays. PWT stands for peptide welding technology, and it is a relatively novel approach to generate with high yield and purity tetrabranched peptide molecules (Guerrini et al., 2014). PWT derivatives of nociceptin/orphanin FQ and related peptides, opioid peptides, tachykinins, and neuropeptide S were generated and characterized pharmacologically in previous studies (reviewed in (Calo et al., 2018)). *In vitro* these PWT derivatives mimicked the effects of the native peptides with no major changes of affinity, pharmacological activity, potency, or selectivity of action. However, *in vivo* studies demonstrated that PWT peptides are generally characterized by increased potency and, more importantly, long-lasting effects (Calo et al., 2018). Accordingly, in this study, PWT2-Dyn A showed pharmacology very similar to Dyn A, and similar selectivity for kappa over mu and delta opioid receptors. In addition, although caution should be exercised in evaluating PWT2-Dyn A data in the BRET assay (i.e. alteration of RLuc light), PWT2-Dyn A seemed to maintain the unbiased features of Dyn A. Clearly, further studies are needed to evaluate the *in vivo* actions of PWT2-Dyn A, assessing the usefulness of this kappa receptor ligand as a novel research tool.

We synthesized and characterized the palmitoylated derivative of Dyn A (Dyn A-palmitic). In the frame of a previous structure-activity relationship study on nociceptin/orphanin FQ, C-terminus palmitoylation has been identified as the chemical modification leading to the highest increase in G protein bias at the NOP receptor (Pacifico et al., 2020). In the same study, C-terminal palmitoylation was also applied at other opioid peptides including dermorphin, deltorphin A, and Leu-enkephalin, which were tested at mu and delta receptors. Palmitoylated peptides consistently displayed high potency in receptor-G protein interaction experiments while their effects in receptor-β-arrestin 2 interaction experiments were variable: they behaved as G protein biased agonists at NOP and delta receptors and as unbiased agonists at the mu receptor (Pacifico et al., 2020). Results obtained at the kappa opioid receptor with Dyn A-palmitic are therefore only partially in line with the findings mentioned above. In fact, C-terminal palmitoylation of Dyn A generated a kappa selective full agonist, but its potency was slightly lower than that of the native peptide. On the other hand, Dyn A-palmitic displayed a statistically significant bias factor towards G protein at kappa receptors, similar to that reported for palmitoylated peptides at NOP and delta (but not mu) receptors. Thus, the present results corroborated previous findings suggesting that C-terminal palmitoylation is a valuable strategy for altering the signaling properties of opioid receptor ligands, although this is not always associated with increased potency. The mechanism by which C-terminal modifications of peptide agonists for opioid receptors promote biased agonism toward G protein is at present unknown. The discussion of this specific issue and some speculative considerations have been reported in (Pacifico et al., 2020). Dyn A-palmitic could be added to the available research tools for investigating functional selectivity and its potential in drug discovery in the opioid receptor field (Dogra and Yadav, 2015; Gomes et al., 2020; Che et al., 2021; French and van Rijn, 2022).

## Conclusion

The overall results obtained in the different assays with standard kappa receptor ligands are in line with literature findings both in terms of rank order of potency of agonists and potency of selective and competitive antagonists. In addition, the use of multiple assays and standard ligands allows detailed pharmacological investigation of activity and classification of ligands as full and partial agonists, neutral antagonists, and inverse agonists. We generated and studied PWT2-Dyn A and Dyn A-palmitic, which demonstrated selective kappa receptor full agonist behavior. Moreover, BRET receptor-transducer interaction studies suggested that PWT2-Dyn A acts as an unbiased agonist while Dyn A-palmitic as an agonist biased toward G protein. Further studies should assess the features of these novel compounds *in vivo* in animal models of pain and pruritus, conditions in which the selective activation of kappa receptors elicits beneficial effects.

## Data Availability

The original contributions presented in the study are included in the article/Supplementary Material, further inquiries can be directed to the corresponding author.
