# Supplementary material for: Pharmacology of Kappa Opioid Receptors: Novel Assays and Ligands
Source: Front Pharmacol. 2022 Apr 21;13:873082. doi: 10.3389/fphar.2022.873082 (PMC9068900; doi:10.3389/fphar.2022.873082)
Supplement: Supplementary file 1 [file DataSheet1.PDF]

SUPPORTING INFORMATION

Pharmacology of kappa opioid receptors: novel assays and ligands

Chiara Sturaro [1], Davide Malfacini [2]✉, Michela Argentieri [1], Francine Medjiofack Djeujo [2], Erika Marzola [3], Valentina Albanese [3], Chiara Ruzza [1,4], Remo Guerrini [3,4], Girolamo Calo' [2], and Paola Molinari [5].

[1] Department of Neuroscience and Rehabilitation, Section of Pharmacology, University of Ferrara, Via Fossato di Mortara 17/19, Ferrara 44121, Italy.
[2] Department of Pharmaceutical and Pharmacological Sciences, University of Padova, Largo Meneghetti 2, Padova 35131, Italy.
[3] Department of Chemical, Pharmaceutical and Agricultural Sciences, University of Ferrara, Via Luigi Borsari 46, Ferrara 44121, Italy.
[4] Technopole of Ferrara, LTTA Laboratory for Advanced Therapies, via Fossato di Mortara 70, Ferrara 44121, Italy.
[5] National Center for Drug Research and Evaluation, National Institute of Health, Rome, Italy.

✉Corresponding author: davide.malfacini@unipd.it

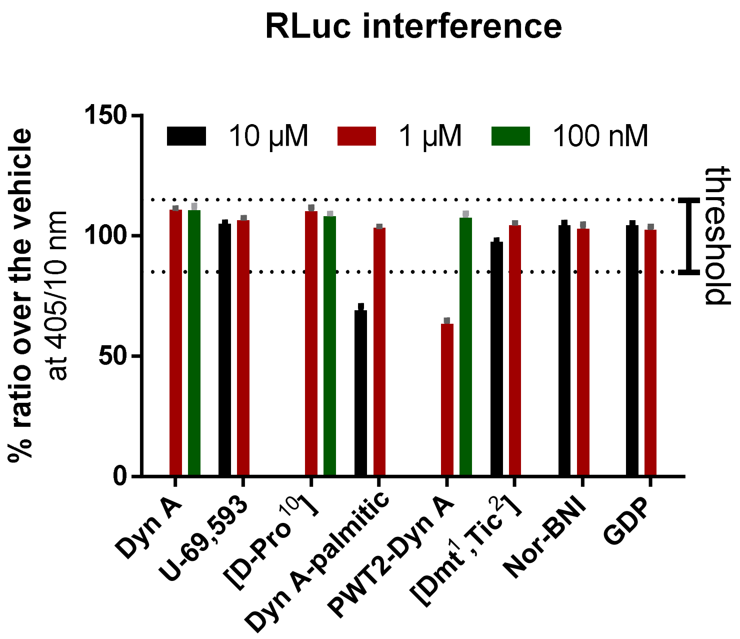

Supplementary Figure 1. Compound interference with the RLuc light. Membranes taken from SH-SY5Y cells stably expressing the human kappa opioid-RLuc tagged receptor together with the β-Arrestin 2-RGFP fusoprotein.

Data are mean + sem of 3 experiments performed in duplicate.

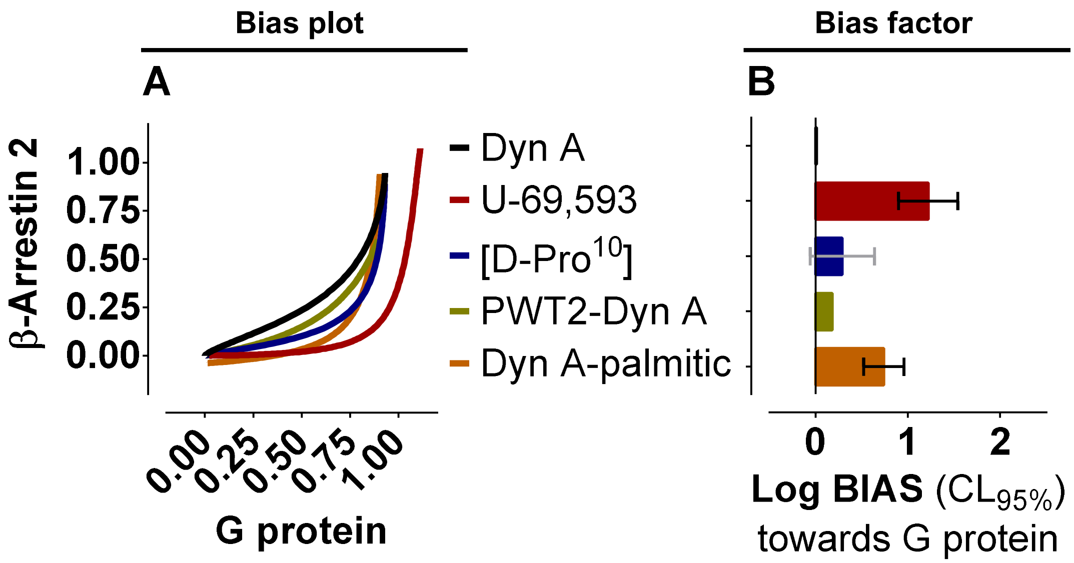

Supplementary Figure 2. Bias plot and Bias factors from G protein and β-Arrestin 2 BRET data.

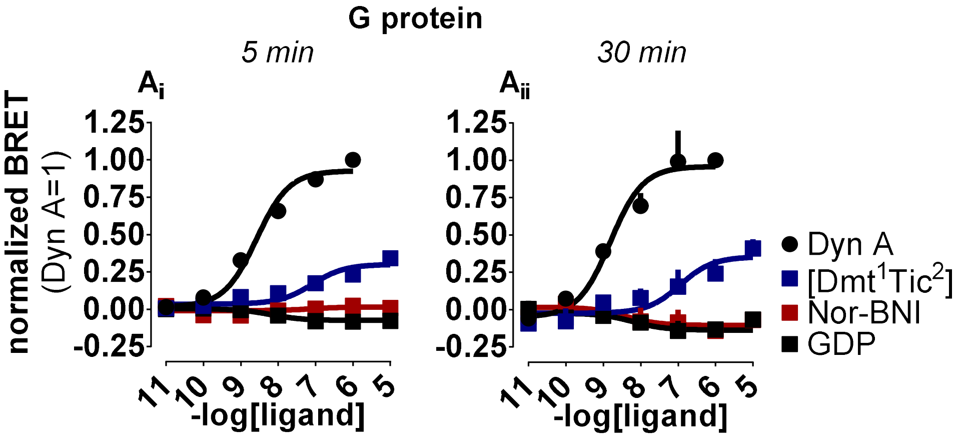

Supplementary Figure 3. BRET experiments on membrane taken from SH-SY5Y cells stably expressing the human kappa opioid-RLuc tagged receptor together with the Gβ_1-RGFP fusoprotein. Concentration-response curves to Dyn A, [Dmt^1,Tic^2], Nor-BNI, and GDP. BRET measurements were made out 5 min (**Ai**) and 30 min (**Aii**) after compound addition. Data shown are mean + sem of at least 3 independent experiments performed in triplicate.

Supplementary Table 1. Pharmacological parameters obtained at 5 min and 30 min in BRET experiments on membrane taken from SH-SY5Y stably expressing the human kappa opioid-RLuc tagged receptor together with the Gβ_1-RGFP fusoprotein.

	**5 min**		**30 min**	
	**pEC$_{50}$ (CL$_{95\%}$)**	**Emax ± sem**	**pEC$_{50}$ (CL$_{95\%}$)**	**Emax ± sem**
**Dyn A**	8.58 (8.43 - 8.72)	1.00	8.81 (8.45 - 9.17)	1.00
**[Dmt^1,Tic^2]**	7.09 (6.68 - 7.49)	0.30 ± 0.02	6.95 (6.14 - 7.75)	0.35 ± 0.06
**Nor-BNI**	inactive		8.40 (6.46 - 10.35)	- 0.11 ± 0.04
**GDP**	8.20 (7.29 - 9.11)	- 0.07 ± 0.01	8.21 (6.41 - 10.01)	- 0.14 ± 0.03

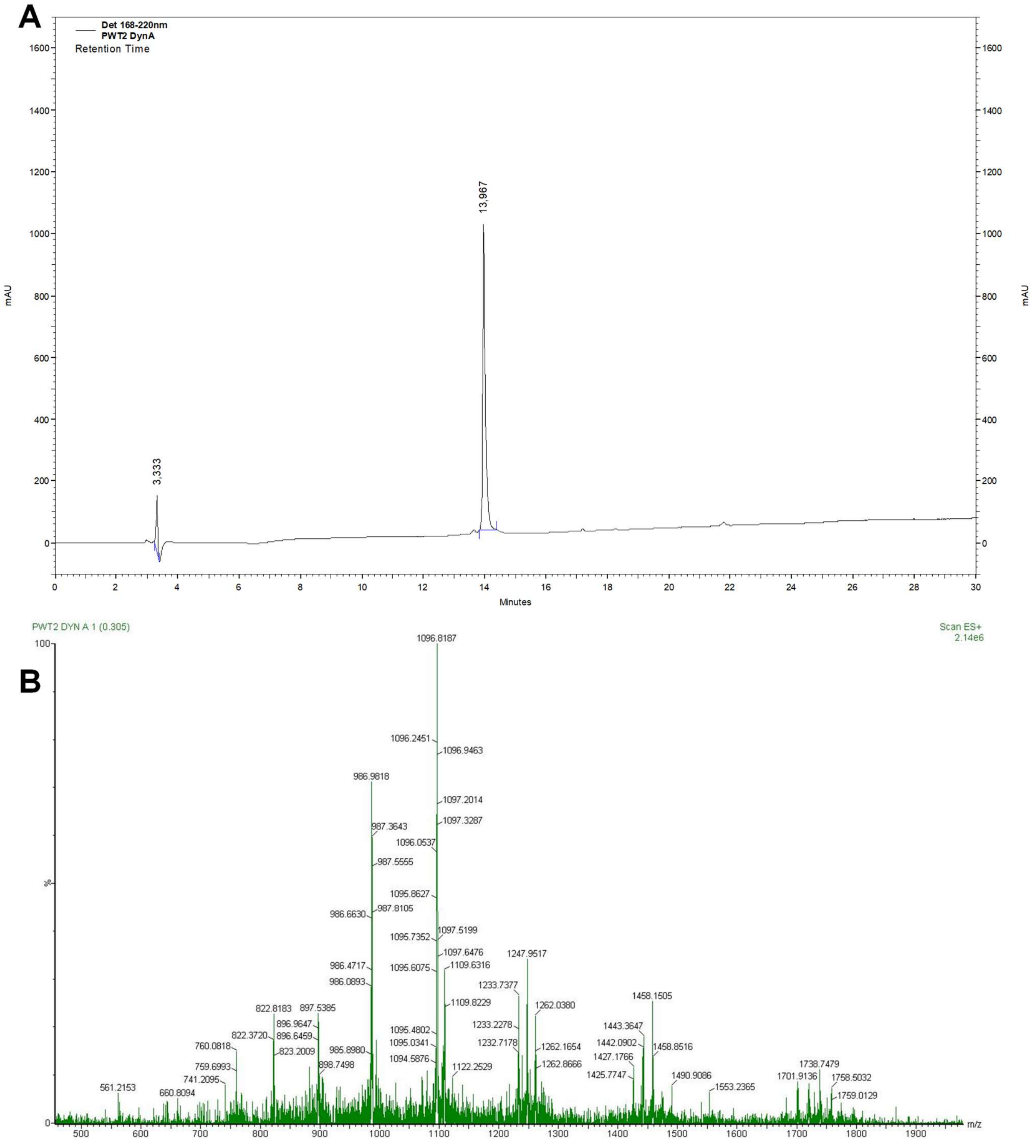

Supplementrary Figure 4. Analytical HPLC chromatogram (**A**) and Electrospray ionization (ESI) mass spectra of PWT2-Dyn A; MW calculated Da: 9859.48 (**B**).

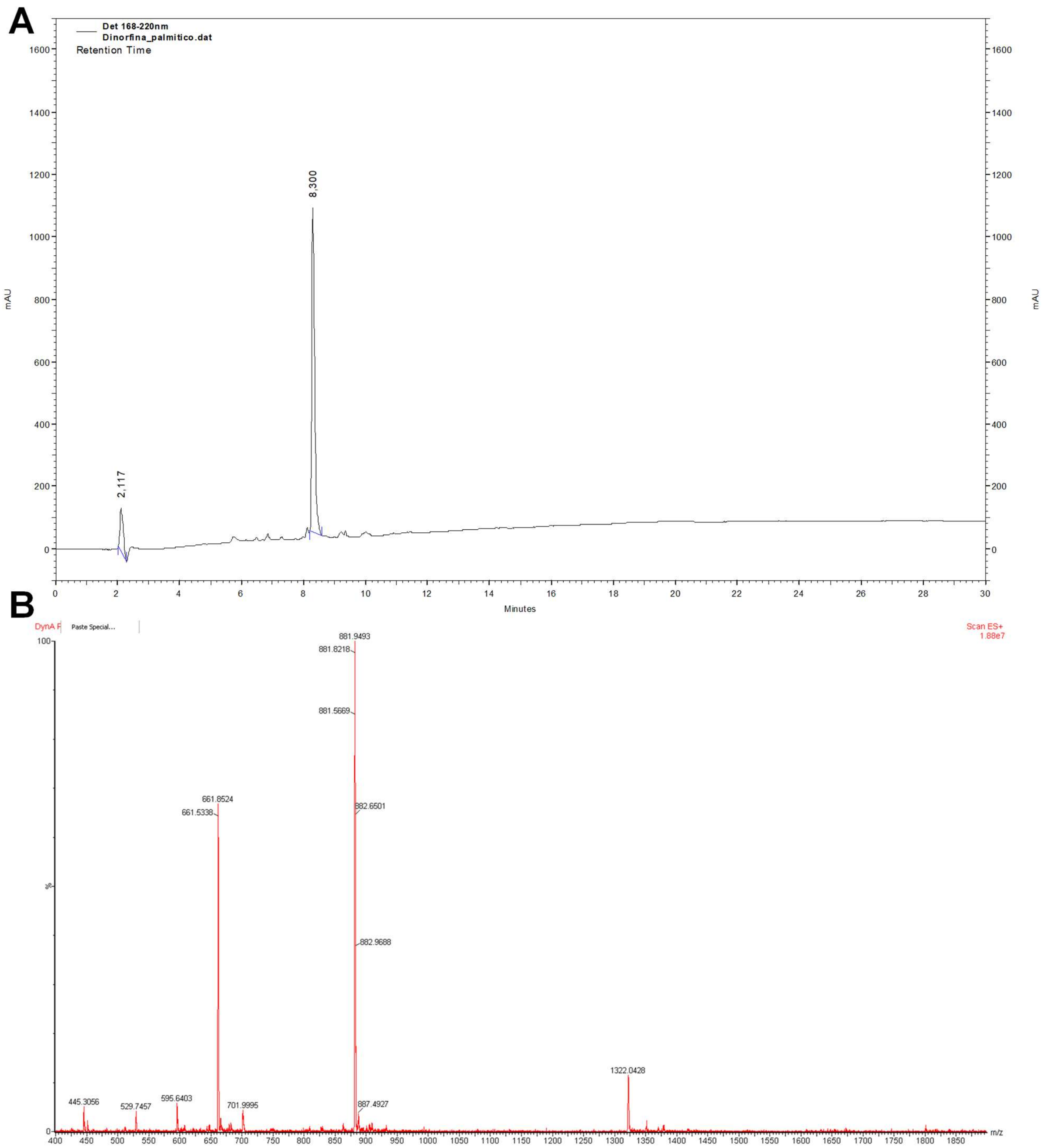

Supplementary Figure 5. Analytical HPLC chromatogram (**A**) and electrospray ionization (ESI) mass spectra of Dyn A-palmitic; MW calculated Da: 2642.26 (**B**).
